# Lessons from the business sector for successful knowledge management in health care: A systematic review

**DOI:** 10.1186/1472-6963-11-173

**Published:** 2011-07-25

**Authors:** Anita Kothari, Nina Hovanec, Robyn Hastie, Shannon Sibbald

**Affiliations:** 1 School of Health Studies, The University of Western Ontario, Labatt Health Sciences Building, Room 222, London, ON, N6A 5B9, Canada; 2 Canadian Institutes for Health Information, 495 Richmond Road, Suite 600, Ottawa, Ontario, K2A 4H6, Canada

## Abstract

**Background:**

The concept of knowledge management has been prevalent in the business sector for decades. Only recently has knowledge management been receiving attention by the health care sector, in part due to the ever growing amount of information that health care practitioners must handle. It has become essential to develop a way to manage the information coming in to and going out of a health care organization. The purpose of this paper was to summarize previous studies from the business literature that explored specific knowledge management tools, with the aim of extracting lessons that could be applied in the health domain.

**Methods:**

We searched seven databases using keywords such as "knowledge management", "organizational knowledge", and "business performance". We included articles published between 2000-2009; we excluded non-English articles.

**Results:**

83 articles were reviewed and data were extracted to: (1) uncover reasons for initiating knowledge management strategies, (2) identify potential knowledge management strategies/solutions, and (3) describe facilitators and barriers to knowledge management.

**Conclusions:**

KM strategies include such things as training sessions, communication technologies, process mapping and communities of practice. Common facilitators and barriers to implementing these strategies are discussed in the business literature, but rigorous studies about the effectiveness of such initiatives are lacking. The health care sector is at a pinnacle place, with incredible opportunities to design, implement (and evaluate) knowledge management systems. While more research needs to be done on how best to do this in healthcare, the lessons learned from the business sector can provide a foundation on which to build.

## Background

The area of 'knowledge management' (KM) emerged in the early 1990s within various fields, including business administration, public policy, information systems management, library and information sciences. KM is viewed as a way of providing the right information, to the right person, at the right time, with the potential of attaining greater competitive advantage [1-5]. Recently, the health care sector has also begun to focus on the systematic management of knowledge [6].

Health care organizations, as late adopters of the KM concept, are starting to implement and evaluate KM strategies [7-9]. Current KM practices in health care are focused on the use of information and communication technologies (ICT)[6,8,9]. Examples of such systems include electronic libraries (e-libraries), repositories containing research articles, clinical guidelines or best practices to assist organizations in managing knowledge [10-14]. A criticism of this approach is that these ICTs are static and do not provide appropriate context to make an effective clinical diagnosis [8]. Further, they do not support knowledge development and sharing. Communities of practice knowledge-sharing strategies have been used to promote interactions among health practitioners. These strategies can be ICT based [7], narrowly focused on practice improvement and/or broadly defined as networks involving multiple stakeholders and objectives [9]. Research has indicated that there may be value in having a venue, or a social space that enables and encourages knowledge sharing to take place [15-17]. Nevertheless, sustainability of such structures continues to be an issue [9]. Another aspect of the health care environment is the strong evidence-based medicine movement that has penetrated continuing education and quality improvement efforts. The point to note is that evidence-based practice focuses on the transfer of explicit knowledge (i.e., research literature), while KM promotes the transfer of explicit and tacit knowledge [7,9]. Tacit knowledge can be described as knowledge that is acquired through practice and experience and can be difficult to communicate (sometimes referred to as "know-how"), while explicit knowledge is often more formal, codified in writing and seen to be easier to communicate [3,18]. A few health care researchers [16,17,19-22] have examined the importance of tacit knowledge, evaluating the role that it plays, and how it ought to be considered in future research. The on-going emphasis on explicit knowledge would have to change if tacit knowledge were to be seen as an important resource in health care. A final observation is that KM health initiatives tend to focus on one solution (e.g., ICT, evidence-based practice) instead of a comprehensive strategy. Overall, there is an increased interest in the health care literature about the importance of capturing, sharing, and using explicit and tacit knowledge within the daily work of health professionals. However, a predominant number of published research articles within the health sector tend to focus on the conceptual and theoretical aspects of KM that, although valuable, lack a pragmatic component.

What makes improvement in KM practices difficult for health care organizations is that much of the advances in KM practice are reported in the business literature. The purpose of this review was to identify and summarize previous studies from the business literature that explored specific KM tools, with the hope of learning lessons from business that could be applied in health care. We also aimed to identify some of the barriers and facilitators encountered in trying to implement a KM strategy.

## Methods

### Literature Search

Business studies were identified by searching seven electronic databases (Scopus, ProQuest ABI/INFORM Global, Proquest Dissertations and Theses, ProQuest Psychology Journals, PsycInfo, and Emerald Library). The search, conducted by a health sciences librarian, was limited to English-language studies published between 2000 and 2009. Search terms were categorized within three overarching themes or concepts, including: knowledge management, organizational knowledge, and business performance. The specific search terms used include: knowledge management (information management, management science, knowledge management solution design, knowledge management systems, knowledge sharing, knowledge management implementation); organizational knowledge (organizational design, organizational change, organizational learning, knowledge transfer, tacit knowledge); and business performance (business outcomes, strategic planning, community of practice, success factors, analysis, failure, assessment, evaluation). Two reasons supported the decision to target search terms in abstracts. First, authors use relevant words in abstracts due to limited space availability, and second, this strategy ensured that identified articles were the main topic of discussion.

Inclusion criteria included studies that (a) contained information about specific KM initiatives (i.e. strategies, tools, and or frameworks); (b) described at least one of the following: type of KM initiative; process involved in the implementation of the KM initiative; evaluation of previously implemented KM initiative, facilitators and/or barriers associated with KM; or lessons learned from previous KM initiatives. We did not include articles published in languages other than English, nor did we include abstracts or unpublished studies. Hand-searching was not conducted. We did not make an effort to contact authors and as such may be missing some articles that are in press. The list of the papers we included in this review (with duplications removed) is found in Additional File 1: Summary of Knowledge Management Studies Derived from the Business Literature (2000-2009) as an additional file; the list of those excluded is available from the first author.

### Selection Process and Data Extraction

Titles and abstracts were screened independently by two reviewers (AK and SS). Articles deemed relevant underwent systematic data extraction, using a data extraction form, independently by two reviewers (NH and RH) to identify overarching themes. Most articles presented theories, or used case study, grounded theory or ethnography methodology. It was decided that critically assessing the quality of the methods used within each study was less helpful than gaining an overall picture of the field and extracting key messages in the fashion of an integrative review.

## Results

The search strategy identified a total of 169 articles. Eighty-six studies did not meet the inclusion criteria, leaving 83 studies in this review. Additional file 1 summarizes the studies.

### Reasons for KM

Previous researchers suggested a number of reasons why an organization might need to consider a KM initiative, including: to help prevent possible knowledge loss (e.g., someone leaving the organization, turnover, retirement)[23-32]; to gain a greater competitive advantage [31,33-35]; the reorganization of the company [31,33-36]; as a formal remedy of negative findings discovered during an audit [37]; continuous learning [38]; to prevent low knowledge diffusion and/or the isolation of organizational departments, individuals, or community partners [25]; to coordinate with other firms/suppliers/customers [39]; to increase the quality of professional services,[40]; and to help meet users' needs [41]. Although specific reasons may vary from one organization to another, a general consensus was that KM can contribute to these sorts of organizational improvements, as well as address an array of intra-organizational problems.

### KM Solutions/Strategies

KM strategies identified from the reviewed literature included: using simple mechanisms (such as training programs and seminar series), using technology, using frameworks or process-based models (including concept mapping), and using communities of practice to capture and share knowledge.

***Simple mechanisms***, including training sessions, workshops, mentoring/apprenticeships, and interviews, were implemented in 6 studies [2,24,28,32,42,43]. Some of the training programs were virtual or web-based [44], however, others were delivered face-to-face because many believe this to be an essential component of training programs [45]. Implementing a seminar series was another dominant KM approach; for example, a specialized "Leaving Expert Debriefing", where managers and/or employees are interviewed by their peers with the goal of retaining the knowledge of "experts" who are planning to leave their current organization [28]. Other seminar series included a number of classes where group work, problem solving, and coaching occurred in interactive settings [28,32].

Despite the various definitions of KM, almost everyone agrees on the significant role technology has in KM. In fact, KM is frequently positioned as being comprised mainly of efficient and effective information technology (IT) and ICT systems [26,27,44,46-48]. For this reason, using ***technology systems ***(and communication technologies) is a key element that may be incorporated into KM initiatives. While details of each technical system vary, the overarching purpose of technology systems is to organize, codify, distribute, and maintain knowledge resources [44]. The predominant focus of many KM strategies is on technology and management of explicit and tacit forms of knowledge.

Other organizations described in the literature designed KM strategies around conceptual ***frameworks or process-based models***[1,2,31,37,40,49-59]. The majority of these frameworks/models included stages to attain, or replicate KM strategy development. One such study featured a roadmap that included an *inner layer *(the KM system backbone: the strategy, sharing, storage, identification, and audit of knowledge); a *middle layer *(necessary success factors: business-process re-engineering, piloting strategies, organizational structure, training programs); and an *outer layer *(factors for successful establishment of all systems in the organization: organizational culture, CEO/executive support, transparency, and trust)[50].

Another framework introduced the concept of ***mapping ***out knowledge, routines, capabilities, and inertia as a tool to advance KM in an organization or unit. The fundamental idea behind this is that latent resources (dormant, but capable of development) are mobilized by endogenous (managerial) or exogenous (legislative/environmental) elements, and that different ratios of active to latent resources, routines and capabilities can have different degrees of adaptability. In stable contexts, organizations that are more adaptable will also be more flexible and responsive, whereas less adaptable organizations are more "lean" (or efficient) but have a limited portfolio of resources to draw on (i.e., more prone to the status quo)[60]. Others have used the mapping concept (e.g. "Capabilities Map", and "Levels of Learning Progression Map") as a process that can capture knowledge-oriented practices [61].

Another concept often considered a useful KM strategy, and heavily discussed in the literature, is ***Communities of Practice ***(CoP)[62-69]. Communities can vary in format (virtual/face to face), and Chua [67] argued they have three main underlying structures:

• *Domain *(the sphere of knowledge and expertise held by members)

• *Community *(relationship, affinity, and the sense of belonging among members)

• *Practice *(the common set of frameworks, ideas, and tools members share in their work context)

The objectives of the CoP should be aligned with the host firm's strategic purpose [63]. Each CoP should have a *Leader *or *Moderator *who spearheads defining the objectives of the CoP and maintaining the focus of the community [65,68]. In addition, there is a *Core Team *who assists the Leader by developing activities and workshops in collaboration with the Leader [68]. The active members each bring their own role, knowledge and expertise to the community [65,68].

Communities may extend past institutional boundaries through online CoPs, an especially important strategy for small organizations to extend their reach [63]. However, it may be difficult to create and sustain online those CoPs with the purpose of knowledge creation as the technology and social structure involve much effort [65]. Moreover, organizations may experience less control over online CoPs outside of their organizational boundaries [64]. Therefore, online CoPs may not be appropriate to address every organization's KM needs.

The literature demonstrates that a one-size-fits-all KM solution may not be achievable or desirable. Organizations may need to invest some time to identify their needs and reasons for employing KM practices, and to ensure that they have the resources (e.g., financial, personal, technical) to support their desired strategy. Identifying possible KM facilitators and barriers may assist various organizations in recognizing whether they are capable of successfully implementing a KM solution or strategy.

### KM Facilitators and Barriers

Since most of the studies included in this review were based on a single case of KM, it is inappropriate at this point to come to any conclusion about which strategies are more effective than others. Instead, we present the common facilitators and barriers to KM initiatives that surfaced across studies.

#### KM Facilitators

Commonly cited KM facilitators include: organizational culture, organizational structure, management and champion support, design of KM strategy, performance/evaluation, and training, which are all further explained in this section.

Thirteen studies indicated that KM can be facilitated by an ***organizational culture ***that is horizontal or flat in structure, with very few or no hierarchy levels [5,29,36,40,62,68-75]. These same studies also put forth that an organization should emphasize the importance and value of people as a main resource, encourage teamwork, and enable knowledge sharing. Further, it is argued that there needs to be a 'knowledge creating and sharing culture'[76,77] with trust and openness at the organization's core [5,35,44,78-81]. Organizations that have shared common values and culture have an advantage when implementing a knowledge management system (KMS)[6]. It is beneficial to use cross-functional organizing, that is, to draw upon expertise regardless of where it may lie within the organization [35]. Physical attributes, such as the configuration of the work environment (i.e., close desk proximity has been associated with more knowledge sharing) influences the knowledge sharing culture in organizations and can contribute to the KM initiatives' success [82]. Also, the delivery channel (for example, intranets, e-mail, internal magazines, meetings, notice boards, etc.) should be selected based on its suitability in relation to the organizational culture of the company [76].

***Organizational structure ***is closely associated with organizational culture and structures and can facilitate collaboration [81]. There is currently not unanimous agreement or understanding of a Human Resources (HR) department's role in KM; for example Oltra found that the HR department should be separate from the KM system [70], while Hsu found it an asset to have HR involved in the development of a KM system [29]. In other words, HR involvement may be fundamental, or completely unnecessary to KM development, depending on the organization's structure, needs, goals and preferences. Oliver and Kandadi suggested allocating appropriate amount of time for employees' learning, collaboration, knowledge creation, and sharing activities as a fundamental structural consideration [82]. Some authors suggested that organizations may require a whole new organizational structure, which would have conventional structures transformed to support a knowledge culture [82,83]. For example, it might be important for an organization to develop a common language (or, an 'organizational thesaurus') to ease the communication within the organization [83].

Many of the studies indicated that ***management support***, including a strong, consistent, and more importantly, cohesive promotion of KM is important to the success of a KM system [25]. Executive involvement and support needs to be built into the KM initiative in order to ensure success [2,5,40,44,46,61,62,67,72,74,76,79,81,84]. Having a ***champion ***(i.e., a very influential individual within an organization who supports KM)[23,28,85,86] in combination with strong KM leadership [29,76,82] was frequently acknowledged as an important facilitator. Recruiting employees with a positive attitude towards knowledge sharing and team dynamics [82], and developing relationships with pertinent individuals [37], is important for successful KM. For example, Hofer-Alfeis argues that to bridge the gap during a transition of power, it is important to promote a relationship between the leaving expert and their successor [28].

Another facilitator for successful KMS was a clear and concise ***KM framework or design***. Plessis noted that the KMS should be linked to the business strategy, and the approach to KM should be holistic with flexible structures, and adaptable to the business environment changes [76]. Human-related factors, or interpersonal interactions, such as face-to-face contacts and close physical proximity, are fundamental to the success of KM initiatives [5,69,80,87-89]. The KMS needs to include processes to help provide standards for KM initiatives and ensure roles and responsibilities within the initiative are clearly defined [76]. Many successful KM systems integrated monetary and non-monetary incentives (such as rewards and recognitions) to encourage the implementation and adoption of KM initiatives [5,25,47,69,75,76,81,82]. Managing knowledge throughout its lifecycle is important (for example, knowledge repositories regularly updated, improved, and fostered in a way to improve decision making)[76] and the process of knowledge sharing should occur continuously throughout the time of employment, not just when someone leaves the organization [29]. Further, the collection of both explicit and tacit knowledge was cited as a KM facilitator [76].

***Training ***that provides a complete and in-depth understanding of how KM works may motivate employees to assist in KM strategy development [40,76,90]. It is especially important that employees are trained in how to use supporting technologies, especially for KM initiatives with a predominantly IT-based focus [81]. Also, employees need time following the implementation of a new KM initiative for reflection and learning purposes [42].

Other facilitators included: perception of the KM system being important, along with encouraging buy-in at all levels by promoting KM as a strategic initiative [76]; recognition of the impact of communication (structured, formal, and informal)[76]; on-going motivation for the KMS [45]; the presence of a clear positive benefit [81]; the quality of the knowledge exchanged [81]; a process based on realistic expectations [25]; and KM has to be cultivated and nurtured (i.e., not a push strategy or a coercive task)[25].

#### KM Barriers

The barriers discussed in the 83 studies can be classified as ***individual ***or ***organizational ***barriers.

At the ***individual level***, change, whether in management, ownership, or employee turnover, can be a source of distress and as such a barrier to KM [37,61,85,91]. Information overload can also pose a barrier to effective KM [49,88,91]. Individuals themselves can be barriers to effective KM if they are unqualified, inappropriate authorities (such as individuals who are in a position of power without the appropriate KM training, understanding of its purpose, etc.), resistant to change [6,26,71,84,92,93], or have insufficient technology skills [6,84,93]. Discussing problems, sharing, or thinking out loud may not come naturally to some, and as such can pose a personal challenge [93], which may also be detrimental to a KM system, since knowledge sharing is a fundamental component of KM. Individuals may lack motivation to implement a KM initiative because of minimal incentives/rewards, time, or desire [5,49,78,79,87,91]. In addition, the loss of a KM champion can devastate the initiative [84]. Lack of support from management and/or employees can pose as a significant barrier to full participation of employees [6,45,50,51,67].

Barriers to KM implementation and success at the ***organizational level ***include both organizational culture and structure [5,25,46,51,60,62,68,69,71,79,94]. More specifically, a top down approach [26], separate departments [31,40], lack of "ask why" thinking [35], lack of trust [6], and not being open to sharing knowledge and information such as "lessons learned"[34] can impede KM efforts. The time and money that it can take to implement a KM initiative may discourage employees from even attempting to develop a KM system [23,50,85,95,96]. Furthermore, it can be difficult to present an observable (or immediate) benefit (i.e., it may take a long time to really see the positive changes contributed by the KM strategy), which may result in the KM concept being viewed as not valuable or not worth the effort/resources needed [74]. For an organization that is heavily invested in technologies, barriers to successful KM may include inconsistencies, malfunctions, or software incompatibility, as well as the challenge of obtaining the software for the knowledge base and a lack of balance between IT and personal interaction [23,30,37,51,68,78,87,88,97,98].

The intangible element associated with knowledge (e.g., "managing stuff in people's heads"), and the nature of organizational learning [5,61,66,92], along with having no standard KM definition are all serious barriers to the success of KM initiatives [23,30]. A lack of clarity about the measurement of KM processes can also hinder KM initiatives [44,45,61,68]. For example, having little or no clear strategy or guidance on how to capture and store important explicit knowledge, or how to translate tacit knowledge into explicit knowledge, or how to evaluate the initiative can present a serious barrier to effective KM [25,30,99].

Disagreement or conflict can present a challenge for KM. For example, if employees have differing goals, or goals that are at odds with the overall KM purpose, this can lead to resentment between middle management and front line workers (for example due to increased work load from KM implementation)[6,23,34,35].

Additional barriers identified less frequently in the review of the literature included concerns regarding implementation of KM initiatives. One such concern includes ensuring enough time (i.e., approximately one year) prior to planning a KM processes and beginning the search for a successor, commencing the KM process (e.g. leaving expert debriefing) early, and hiring a successor before starting the KM strategy allowing him/her to learn from the leaving expert [28]. Furthermore, monetary rewards can potentially create unfriendly team environments, resentment, competition and loss of focus of the overall KM purpose [25]. Also, one should be aware of over-management and interference from the political sphere trying to force KM to occur prior to considering a KM initiative [6].

To summarize, some individual-level barriers can be overcome, such as training or allocating adequate time for KM work. Organizational environments that downplay reporting hierarchies in favour of openness and a shared culture are more favourable to KM strategies. While management support is crucial for success, the need for support from human resources departments is not conclusive. Another important consideration is a clear KM framework or strategy that incorporates human factors (e.g., rewards, face to face time). Finally, the challenges that accompany IT need to be addressed - for example, rapidly evolving technology that demands adaptation, or difficulties in using IT infrastructure in a way that is appropriate for the organization's needs [8].

## Discussion

Interest in KM has increased in fields outside of business, particularly in health care, where health practitioners are beginning to realize the potential of embedding KM concepts in their own practices and organizations [6]. Improvements in business processes, better coordination with other departments or with outside stakeholders and prevention of information loss due to staff retirements are cited as important reasons for turning to KM practices in the business sector. Thus, the catalyst has been described as a desire for organizational improvements, and not so much as staff-level advancement or professional development. Although there has been a marked increase in academic publications related to KM over the past ten years, there are unanswered questions about which strategies are most effective given that direct comparisons have not been studied systematically. The reviewed literature discusses passive 'push' strategies to sharing knowledge, e.g., seminars, as well as the ways in which technology has been used to encourage asynchronous interactions among workers. Social learning strategies in the form of communities of practice were commonly identified. Finally, the literature reviewed pointed to systems perspectives in the use of frameworks (e.g., capabilities maps). While the common business solutions have been reported here, several other tools are also likely to exist which have not been published, or are not easily accessible. The facilitators and barriers related to implementing KM solutions in a business environment were also raised in this review. These facilitators and barriers were multilevel (individual, unit, organization), and inter-related (for example, individual motivation is related to organizational rewards, structures and learning culture). A key learning that emerges is that organizational context is an important consideration in the application of KM approaches, as organizational structures and processes contribute to the ability of an individual to carry out knowledge sharing activities that are sustainable.

Before discussing how the lessons from the literature might be applied in health care, it is worth noting the ways in which the health sector differs from the business sector in terms of organizational context. Health care organizations tend to be under-resourced, and they are expected to perform in accordance with state or national health policies, while private sector organizations are responding primarily to internal goals. Related to this, health care organizations are more likely to run into political interference (or support) by elected officials than an independent business might experience. Health care organizations are often the linchpin holding together collaborations with other health agencies and civil society organizations; through these inter-organizational arrangements, information and practices are shared to support a continuum of care in the community. In contrast, in the business sector key information is withheld in service of a competitive advantage in the marketplace. Business is focused on profit, while health care aims to produce a somewhat intangible public good. Inside a health care organization one is likely to find different professional groups who belong to different unions; are paid through different funding envelopes (e.g., hospital budget or reimbursed through the state); are paid through different funding mechanisms (e.g., salary or fee for service); and who have strong alliances with their professional community across organizations. Within an organization, these different groups exhibit a particular professional culture. Despite these differences, however, both sectors experience the common influences of new technology, globalization, operational optimization and the need to evolve through reforms and transformation [100].

Thus in discussing ways that health care organizations might move forward with a knowledge management agenda, it is acknowledged that there are variations across organizations and that sensitivity to contextual conditions is vital. These differences are important for understanding "how context and purpose may shape learning strategies, processes and outcomes"[100]. Nevertheless, KM experiences from the business sector can contribute to advancing the current KM status quo in health care. The health care environment, described in the introduction of this paper, can be characterized as such: 1) ICTs as current KM strategies in the health arena are static and do not support knowledge sharing, 2) Communities of practice and networks, as another popular KM strategy in the health arena, require attention in terms of long term sustainability, 3) the dominant evidence-based culture stresses research information, and as a consequence, less attention is devoted to tacit knowledge, and 4) KM strategies in the health arena tend to be single initiatives, which may limit effectiveness and sustainability. Below we elaborate on how to move forward in these four areas.

Experiences from business point to examples of ICTs for knowledge sharing purposes, such as wikis or blogs. These technologies can help support knowledge management and e-learning by enabling users to access content of interest quickly and conveniently. Further, interactions between individuals can also serve to co-create new, relevant knowledge. Some authors have suggested that KM and IT advances can have a strong and beneficial impact on the quality of health decision-making [13]. To transfer this learning successfully into the health realm, it would be important to identify non-hierarchical groups, such as a professional discipline, who might readily share best practices with each other. Alternatively, such strategies might be ideal for multidisciplinary care teams who provide care collectively and share a similar culture. In this way, technology - serving as the common boundary object across professionals - can help in the creation and support of virtual communities to help maximize the sharing of knowledge and learning [7]. Not to be forgotten is the patient population: patients are being invited to participate in their own care through shared decision making, and ICTs can play an important role in facilitating access, discussions and understanding of complex medical and health information.

There is documented interest in communities of practice and networks in health; these structures are perceived as a new way to organize public services [101-104], but their long-term viability is of concern [9]. Experience from the business sector suggests that "one-size fits all" or externally imposed programs may lead to the underdevelopment of knowledge and/or limited sustainability. One study in particular perceived communities of practice to be the key to a successful KM initiative [66]. In this study the authors reported that communities of practice strengthen topic-specific social networks by enabling knowledge retention and allowing for the dissemination of best practices and lessons learned [68]. In addition to a common topic of focus, sustainability might be achieved by ensuring that online networks incorporate a face-to-face component for community-building purposes. Business leaders are demonstrating that they value their employees' tacit knowledge - their employees' experiences and interpretations derived from interacting with the company and those associated with the company - by devoting resources to capturing tacit knowledge. The business sector has moved from simple repositories of such information to more active approaches, knowing that sustainability requires an interactive approach to managing knowledge. Engaging workers in CoPs or networks helps build the collective knowledge base (or 'knowledge capital') and expand knowledge assets, which in turn will help foster a sustainable organizational context.

The area of evidence-based medicine, a paradigm of clinical teaching and practice, might be a deterrent to the use of KM practices in health care. Dedicated journals, practice guidelines, research use frameworks, and supporting organizations promote the use of medical research literature, leaving little room for the sharing of tacit knowledge, an important foundation of KM strategies. The evidence-based medicine movement has spread to other areas of health care. The broader field, now referred to as knowledge translation, has developed from efforts to explain and promote the use of research evidence in clinical, managerial and policy decision-making related to health care [105-107]. Both the evidence-based medicine and knowledge translation movements have encouraged health care professionals and their organizations to seek out relevant research evidence and adapt it for internal decision-making for the eventual purposes of improved health outcomes. Rather than a deterrent, however, this paradigm might be seen as an opportunity with which to introduce KM practices. Health care professionals have already been engaged with the notion (and related techniques) of using externally derived information in a systematic way. Using KM strategies to promote the use of organizationally-based, internally derived information should be seen as a natural extension of knowledge translation. We suggest that knowledge management can overlap with the knowledge translation process, with the merger occurring when local knowledge (e.g. tacit knowledge [1,2] or data such as a local needs assessment) is used in conjunction with research evidence.

As this is a relatively new area for health managers and executives, KM initiatives have been, generally speaking, designed as single-faceted interventions. The review describes KM as an interpersonal and an organizational process, and as such both may influence the success of implementing a KM initiative. Multi-faceted interventions can support implementation efforts by: addressing the organizational and individual limitations described in this paper; indicating upper management support for KM; and providing a carefully planned but flexible approach for the organization. The reviewed literature of KM in the business sector suggests that a holistic or multilevel KM strategy may be useful for health care organizations to enhance embeddedness. Multiple, coordinated initiatives are required to achieve a synergistic use of ICTs with new approaches to linking people with information, and research with data, and supporting the conversion of information and data into useable knowledge.

There is still a fair amount of research to be done in this area, especially in the health care sector. Moving forward, a common set of definitions and dimensions will enable health care practitioners to better share information and promising KM strategies in order to build momentum in this rapidly expanding field. At the very least, health researchers ought to include a comparison group in their KM studies. Longitudinal research related to KM and culture change would also be welcome. Trying to understand current KM practices (e.g., to identify what is being done currently across health organizations in terms of knowledge capture and sharing), to test potentially effective KM strategies for the health care context, and then to implement such strategies across an appropriate level of analysis (e.g., across a department or the whole hospital) are areas that require attention.

In this integrative review we addressed methodological rigour in a number of ways. Two authors (AK and SS) reviewed the abstracts for inclusion, and two other authors (NH and RH) systematically extracted the information from articles using a data extraction form. In both cases the two researchers worked independently and then met to discuss any discrepancies. We intended to critically assess the research process associated with each study but we abandoned this approach given the prominence of case study methods. Searching the literature was a very difficult task due to the diverse and evolving vocabulary. It is therefore possible that we missed some important articles along the way (including non-English ones).

## Conclusions

The area of KM thrives in the business literature as a way to capitalize on an organization's internal knowledge. KM strategies include such things as training sessions, communication technologies, process mapping and communities of practice, and while common facilitators and barriers to implementing these strategies are discussed in the business literature, rigorous studies addressing the effectiveness of such initiatives are lacking. The concept of KM is just beginning to emerge in the health care sector, providing an ideal opportunity to properly evaluate knowledge capturing, sharing and storing systems. Further thought is also required about how to reconcile the concepts of knowledge translation and knowledge management for optimal health care processes and processes, and ultimately, health outcomes.

## Competing interests

The authors declare that they have no competing interests.

## Authors' contributions

AK conceptualized and implemented the study. AK and SS reviewed abstracts and wrote sections of the manuscript. NH and RH extracted data from the articles and wrote sections of the manuscript. All authors discussed the findings and provided critical feedback on draft manuscripts. All authors also read and approved the final version of the manuscript.

## Pre-publication history

The pre-publication history for this paper can be accessed here:

http://www.biomedcentral.com/1472-6963/11/173/prepub

## Supplementary Material

Additional file 1 **Summary of Knowledge Management Studies Derived from the Business Literature (2000-2009)**. Complete list of the papers included in this review (with duplications removed). Click here for file
